# Dealing with context in logic model development: Reflections from a realist evaluation of a community health worker programme in Nigeria

**DOI:** 10.1016/j.evalprogplan.2018.12.002

**Published:** 2019-04

**Authors:** Bassey Ebenso, Ana Manzano, Benjamin Uzochukwu, Enyi Etiaba, Reinhard Huss, Tim Ensor, James Newell, Obinna Onwujekwe, Nkoli Ezumah, Joe Hicks, Tolib Mirzoev

**Affiliations:** a Nuffield Centre for International Health and Development, University of Leeds, Worsley Building, Clarendon Way, Leeds, UK; b Health Policy Research Group & the Department of Health Administration and Management, College of Medicine, University of Nigeria Enugu-Campus, 400001, Nigeria; c School of Sociology and Social Policy, Social Sciences Building, University of Leeds, Leeds, UK

**Keywords:** Programme theory, Realist evaluation, Logic model, Community health workers programme, Maternal and child health, Nigeria, Context

## Abstract

Logic models (LMs) have been used in programme evaluation for over four decades. Current debate questions the ability of logic modelling techniques to incorporate contextual factors into logic models. We share experience of developing a logic model within an ongoing realist evaluation which assesses the extent to which, and under what circumstances a community health workers (CHW) programme promotes access to maternity services in Nigeria. The article contributes to logic modelling debate by: i) reflecting on how other scholars captured context during LM development in theory-driven evaluations; and ii) explaining how we explored context during logic model development for realist evaluation of the CHW programme in Nigeria. Data collection methods that informed our logic model development included documents review, email discussions and teleconferences with programme stakeholders and a technical workshop with researchers to clarify programme goals and untangle relationships among programme elements. One of the most important findings is that, rather than being an end in itself, logic model development is an essential step for identifying initial hypotheses for tentative relevant contexts, mechanisms and outcomes (CMOs) and CMO configurations of how programmes produce change. The logic model also informed development of a methodology handbook that is guiding verification and consolidation of underlying programme theories.

## Highlights

• We describe the process of developing a logic model as part of an ongoing realist evaluation of a community health workers programme in Nigeria.
• We also reflect on how other scholars explored context during logic modelling in theory-driven evaluations of complex healthcare programmes.
• Creating a logic model is a key step in modelling relations between a programme, its outcomes and contextual conditions that can affect outcomes.
• Logic models can also inform identification of initial working theories and evaluation measures for verifying underlying programme theories.
• Lessons for researchers and programme planners who wish to develop and use logic models in future evaluations are discussed.

## 1 Background

There have been growing calls, in the past four decades, for programme planners and service providers to describe the intervention models and underlying theories of their programmes to demonstrate how programmes will facilitate change (Davies, Walker, & Grimshaw, 2010; Powell et al., 2017; Sundra et al., 2006; Yampolskaya, Nesman, Hernandez, & Koch, 2004). Hitherto, many funders require logic models (LMs) to demonstrate that programme strategy and outcomes are grounded in theory, and to facilitate monitoring and evaluation (Helitzer, Willging, Hathorn, & Benally, 2009; Judge & Bauld, 2001; Kaplan & Garette, 2005). Consequently, researchers and evaluators increasingly use LMs for understanding the linkages among programme objectives, inputs, activities and outcomes. Theory-driven evaluation is “any evaluation strategy or approach that explicitly integrates and uses stakeholder, social science, some combination of, or other types of theories in conceptualizing, designing, conducting, interpreting, and applying an evaluation” (Coryn, Noakes, Westine, & Schröter, 2010). According to Rogers (2006), theory-driven evaluation approaches have two essential elements: an explicit model of the programme depicting the assumptions and ideas (i.e. ‘theories’) that inform the making of a programme, followed by an empirical investigation to gauge the extent to which those theories are met within complex contextual circumstances of implementation practices (Manzano & Pawson, 2014). Theory-driven evaluators use several methods with varied levels of detail and complexity for eliciting, “constructing or reconstructing” programme theory, including logic modelling, logical frameworks, outcomes hierarchies and Antecedent Target Measurement (Astbury & Leeuw, 2010; Blamey & Mackenzie, 2007; Renger, Foltysova, Becker, & Souvannasacd, 2015; Rogers, 2008).

Nevertheless, current debates in the LM literature criticise scholars for overlooking the role of context in logic model design, construction and usage (Morell, 2014; Renger et al., 2015) This article contributes to addressing this shortcoming by: i) reflecting on how theory-driven evaluation approaches account for context during LM development; and ii) presenting how we explored context during LM development for realist evaluation of a community health workers’ (CHW^1^) programme in Nigeria.

In the realist evaluation (RE) approach, which is a form of theory-driven evaluation, evaluators do not ask ‘what works?’, ‘does this work?’ or ‘did this work this time (Pawson & Tilley, 1997)?’ Rather, they seek to establish “how and why programmes work (or do not work), for whom they work, to what extent, in which settings and for how long?’(Westhorp, 2014, Pg4). In recognition of the influence of context on programme implementation and its outcomes, Pawson and Sridharan (2010) urged scholars to model, in diagrammatic form, the process through which programmes achieve their ends. In response to Pawson and Sridharan’s call (2010), this paper shares how we developed our LM within an ongoing RE of a community health workers programme in Nigeria, including how we incorporated context into the LM. An overall aim of the RE is to assess the extent to which and under what circumstances, the CHW programme promotes equitable access to quality maternity services in Nigeria and improves maternal and child health (MCH). A secondary aim is to assess the sustainability of achieved outcomes and the effects of ongoing advocacy efforts to entrench MCH in the national political agenda.

The paper begins by explaining the concept of logic modelling and its usefulness to programme stakeholders while reflecting on categories of context to consider when developing or using LMs. After that, we examine how scholars have used logic models to develop programme theories in theory-driven evaluation, including in realist evaluation. We then describe the background to the CHW programme and how we created a LM for our evaluation. The process used to incorporate context into the LM and develop initial working theories are also discussed. We conclude with lessons for those who wish to develop and use LMs in future programme evaluations.

## 2 Literature review

### 2.1 Logic models and their purpose

Logic models are tools for planning, describing, managing, communicating, and evaluating a programme or intervention (CDC, 2013; Millar, Simeone, & Carnevale, 2001; Naimoli, Frymus, Wuliji, Franco, & Newsome, 2014). The LM offers a simplified visual representation of the relationship between various components of a programme (Kaplan & Garette, 2005; Renger et al., 2015), and may include assumptions that underlie expectations that the programme will work under certain environmental conditions to solve a particular social problem (Knowlton & Phillips, 2012; McLaughlin & Jordan, 2015). Logic models vary in their complexity and take many different forms, including flowcharts, tables, pictures, and diagrams, and can include different components (Funnell & Rogers, 2011; Newton, Poon, Nunes, & Stone, 2013; Petersen, Taylor, & Peikes, 2013). The literature reveals at least five ways in which scholars use LMs:

a) **Assessing feasibility of a programme at the inception stage:** A clear and brief presentation of the programme theory at the programme conception stage is vital (Clapham, Manning, Williams, O’Brien, & Sutherland, 2017) for deciding whether the programme: i) has a good chance of being implemented with planned resources, ii) has a reasonable chance of achieving its intended outcomes, and iii) is presented in a manner that can aid planning of its evaluation (McLaughlin & Jordan, 1999; Savaya & Waysman, 2005).
b) **Clarifying goals and conceptual gaps:** A coherent LM can help implementers and evaluators reach consensus about their goals and uncover gaps in programme logic. A collaborative approach to considering goals and conceptual gaps at the programme development stage (Newton et al., 2013) enables stakeholders to clarify, specify or modify resources and activities before full-scale implementation commences (Petersen et al., 2013)
c) **Monitoring progress of implementation and changing needs:** A LM can provide implementers and evaluators with a framework for monitoring how the programme and/or its components evolve over its life span. For example, a LM can help determine whether inputs are sufficient or activities are implemented according to plan, in order to identify areas for modification or provide technical support for ongoing implementation (Petersen et al., 2013).
d) **Developing measures of evaluation:** By outlining the important components and inner workings of a programme, proverbial “black box”, (Petersen et al., 2013), a LM can serve as a focal point for discussions about data collection by informing decisions about key aspects of a programme that should be evaluated and ensuring that evaluators identify indicators of all elements that are critical to programme theory (Conrad, Randolph, Kirby, & Bebout, 1999; Sherman, 2016). Designing data collection based on a LM can aid examination and testing the programme logic and provide a plausible explanation for the hypothesized causal mechanisms if intended outcomes are attained. LMs can also help evaluators identify critical questions that can/should be answered and guide priorities and allocation of resources (Petersen et al., 2013).
e) **Dissemination and knowledge building:** LMs can offer an efficient way of assessing the applicability and generalizability of programmes to other settings or populations, especially as stakeholders often want to see their investments (of time, money and energy) yield benefits beyond the immediate setting of programme implementation (Savaya & Waysman, 2005). Scholars therefore urge evaluators to use LMs to facilitate knowledge transfer from some programmes or sites to others, by explaining the processes that cause or prevent the achievement of intended outcomes (Cronbach et al., 1980; Shadish, Cook, & Leviton, 1991).

### 2.2 Synopsis of debate regarding incorporating context into logic models

Most approaches to creating logic models have focused on simple, linear models, but some have explored how non-linear models might be used (e.g. Funnell, 2000) to better represent programmes and guide their evaluation (Rogers, 2008). While there is broad agreement that LMs are useful for summarizing the “logical’’ process of linking underlying programmatic assumptions, inputs, activities, outcomes and impact, nonetheless, the ability of logic modelling techniques to incorporate contextual factors into simple LMs is contested. While some authors have praised the incorporation of contextual factors in LMs as a strength of logic modelling (McLaughlin & Jordan, 1999; Owen & Rogers, 1999), others criticize LMs for their failure to capture the external context in which programmes operate (Cullen et al., 2016; Hill & Thies, 2010; Rogers, 2008). Moreover, scholars have highlighted an unintended consequence of some simple formats of LMs. For example, Morell (2014) and Renger et al. (2015) noted that the limitation of the table-format of LM is that it only depicts the subset of contextual conditions directly targeted by a programme while ignoring the broader context of the other underlying conditions that contributed to the problem the programme aimed to change. Rogers (2008) argues that, by leaving out the other factors that contribute to observed outcomes, including the implementation context, concurrent programmes and the characteristics of clients, simple logic models risk overstating the causal contribution of the intervention, and providing less useful information for replication. Interestingly, the heterogeneity of LMs (whether simple or complex in format) derives from the different purposes and interrelated contexts in which they may be used. In this sense, two interrelated contexts can be differentiated: the contextual circumstances in which a programme is implemented; and the context within which the evaluation of the programme is conducted. A brief description of these contexts is provided next.

a) **Programme context:** This relates to the programme’s contextual factors e.g. policies, institutional, cultural and socio-economic factors that affect users or deliverers of the programme. Although context is traditionally understood as” factors that are external to and operate outside of a programme’s control but may influence the implementation of the programme” (Cullen et al., 2016), context also permeates across the individual, organisational and system levels (Ebenso et al., 2017; Mirzoev et al., 2016). According to the RAMESES II project (2017), realist evaluation considers the settings into which programmes are introduced as social systems with meanings, rules and sets of relationships that interact with/influence: i) responses of stakeholders to programme resources and ii) intended programme outcomes. This is important as a detailed understanding of contextual factors assists in identifying the challenges and assumptions that affect implementation and, eventually, programme successes and failures. It also increases understanding of how unforeseen and unplanned contingencies can affect programme mechanisms, resources and expected outcomes (Cullen et al., 2016).
b) **Evaluation context:** Over the past four decades of praxis, LMs have been used in many evaluation contexts (Park, Welch, & Sriraj, 2016). Evaluation contexts relate mainly but not exclusively to programme size and type; evaluation purpose; budget and timeframes; stakeholders involved in the evaluation and their interests and values; the evaluation approach (e.g. summative/formative, theory-driven etc.). These components of context all impact the objectives of the selected LM. For example, while some argue that LMs can be applied to all kinds and sizes of programmes (Porteous, Sheldrick, & Stewart, 2002), the application of LMs in large scientific research programmes is often debated (O’Keefe & Head, 2011) with evaluators reporting challenges when using logic models in multi-site community-based programmes (Cullen et al., 2016; Kaplan & Garrett, 2005). Newton et al. (2013) also differentiate between LMs used in evaluations, from those used in social science research studies explaining that the latter tend to isolate variables while professional evaluators acknowledge the complex interactions among program activities and outcomes.

Closely linked to both programme and evaluation contexts are decisions concerning the purpose for developing a LM, the people who develop it and circumstances in which the LM is developed as these are likely to impact the process of and the end results of the LM’s development. These contextual and methodological decisions ultimately influence the format of LM selected, the components of the programme represented in the LM and the language used for describing features of LMs (Moss, 2012). Both types of contexts were taken into consideration and explored during LM development for realist evaluation of the CHW programme in Nigeria. While we differentiate between the programme and evaluation contexts, we acknowledge the apparent overlaps between the two and a possible argument that the distinction between the two contexts may be somewhat less clear. Next, we examine how theory-driven evaluations (including realist evaluation) use LMs to explore context. This is followed by a description of the realist evaluation of the CHW programme in Nigeria and methods we used to develop the LM within the RE of the programme.

### 2.3 Using LMs in theory-driven evaluations

Theory-driven evaluation is a collection of evaluation methods that highlight the significance of understanding the theories underlying a programme approach, before evaluating it (Breuer, Lee, De Silva, & Lund, 2016). As mentioned in the background section, the programme theories are first made explicit and thereafter used to assess how programme activities lead to intended outcomes. Breuer et al. (2016), Pg 2) contend that there are a number of interrelated theory-driven evaluation approaches in existence including: logic models, logical frameworks, outcomes hierarchies and Theory of Change (ToC). Conversely, other evaluators do not see these as distinct evaluation approaches but rather as methods used within theory-driven evaluation for eliciting, (re)constructing programme theory (Astbury & Leeuw, 2010; Blamey & Mackenzie, 2007; Renger et al., 2015; Rogers, 2008). ToC was developed by Weiss (1997) within the tradition of theory-driven evaluation, as an approach for explaining how a programme brings about specific long-term outcomes through a logical sequence of intermediate outcomes (Breuer et al., 2016). Regardless of its similarity to other theory-driven evaluation approaches, ToC has significant differences. For example, whilst LMs are useful for depicting a simplified and linear model of the components (i.e. input, activities and outcomes) of a programme, nevertheless, LMs do not explicitly articulate the causal links through which the programme produces change in the way that ToC does (Breuer et al., 2016). Similarly, although logical frameworks (logframes) have utility in summarising programme resources, inputs, outputs, outcomes, indicators of success and the assumptions/risks to the programme, unfortunately, the inflexibility of funder-driven formats has turned logframes into a results-based management tool, that does not facilitate the articulation of causal pathways through which components of the logframe work together. On the other hand, realist evaluation, focuses on articulating the underlying generative mechanisms that link a programme's processes and inputs to outcomes and contexts (Pawson & Tilley, 1997).

**Fig. 1:**
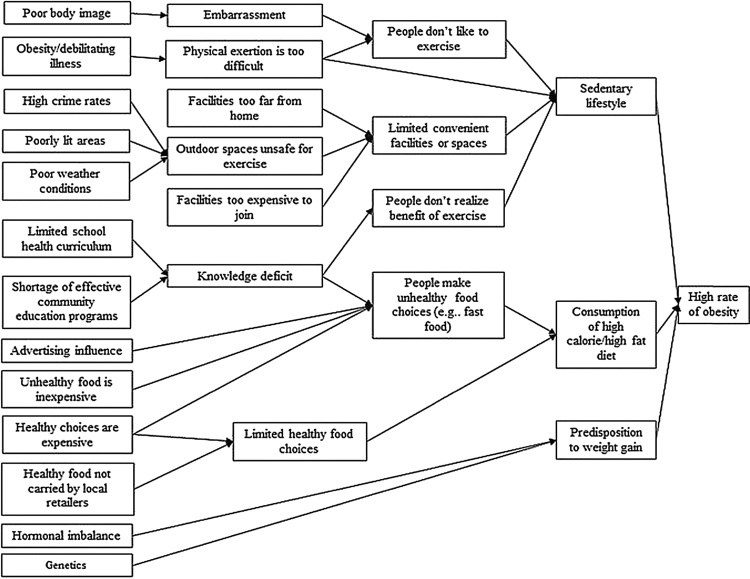
Example of a context map (Source: Renger et al., 2015, pg121).

Evidence shows that simple LMs were favoured in earlier approaches of theory-driven evaluations to enhance implementation and adoption of evaluation findings (W.K.-Kellogg-Foundation, 2004). However, in the last decade, more comprehensive or ecological models have been advocated to help account for multiple and interacting contextual factors that may impact on programme processes and outcomes (Coryn, Noakes, Westine, & Schröter, 2011). These more recent evaluation models tend to start by: i) identifying the detailed specification of underlying processes, to assess whether they have been met and: ii) identifying contextual factors at macro, meso and micro levels that influence their achievement. For example, to address the limitation of the table-formation of LM highlighted earlier, Renger et al. (2015) employed a two-step strategy to adapt the Antecedent Target Measurement (ATM) approach for logic modelling, to facilitate exploration and incorporation of context into their development of LM. Firstly, to generate an understanding of programme context, Renger et al. (2015) engaged programme stakeholders (e.g. programme staff, expert scholars and business executives) in a root cause analysis (RCA) interviews, to identify and visually represent the relationship between the problem of obesity and the underlying conditions that contributed to the problem. RCA interviews entailed delving deeper, by asking stakeholders a succession of “why” questions about underlying conditions related to obesity. The results of Renger et al.’s RCA interviews were then incorporated into a single diagram called the context map. Renger et al. used the context map (see Fig. 1 for example) to systematically and visually depict context by identifying as many of the conditions as possible that contributed to obesity and the relationships between them.

Secondly, following identification of myriads of contexts, Renger et al. engaged key programme stakeholders in a prioritization process (aided by a matrix shown in Table 1), to filter through, and rank the numerous conditions in the context map, to arrive at a manageable number of contextual factors to include in their evaluation.

**Table 1:** Matrix for prioritizing antecedent conditions for outcome evaluation (Source: Renger et al., 2015, pg122).

	High coverage (Two or more targeted underlying conditions)	Low coverage (one activity targeting an underlying condition)	No coverage
High (immediate) control	**High priority** e.g., Healthy food not carried by local retailer	**Medium priority** e.g., Limited school curriculum	Do not evaluate
Low (intermediate) control	**Medium–high priority** e.g., Limited healthy food choices	**Low priority** e.g., People make unhealthy food choices	Do not evaluate
No control	**Do not evaluate**	**Do not evaluate**	Do not evaluate

While Renger et al. (2015) used the context map as a precursor to developing their programme theory and incorporate context in the LM, they acknowledged that the utility of context maps can be augmented by considering the premises of realist evaluation), which underlines the importance of context in interpreting programme outcomes.

However, realist evaluators use LMs in a different way. Rather than gathering as much evidence as is feasible on each component of the LM, to answer the question “Did this happen?’’ about each programme component’, like many evaluation approaches would do, realist evaluations are also interested in “why this did or did not happen? and what circumstances contributed to their happening/or not happening?” (Rogers, 2008). In this sense, LMs are tools for understanding the first evaluation step (i.e. figuring out what is the plan? and the programme theories supporting it) and then presuming that every single link in all the possible series of inputs, outputs and outcomes is (or produces) a possible Context, Mechanism, Outcome (CMO) configuration and that there are an infinite number of such CMOs. This is more than considering the specific contextual factors in which the programme is implemented; it is about elucidating the “social rules, values, sets of interrelationships” that either constrain or support the activation of programme mechanisms and eventually of outcomes (Pawson & Tilley, 1997, p70).

Prioritising which CMOs to evaluate is potentially an endless task, in which anomalies and contradictions are pursued in the hope that tentative causality hypotheses will emerge about how the programme brings about change. Data collection methods are also designed to identify how this ideal model is operationalised locally when confronted with the inevitable implementation challenges and the infinite complexity of open systems and to ascertain where and why programme fidelity succeeded or failed. Besides the ideal model, a second model of intentional or unintentional programme adaptations will emerge in the form of deletions, alterations and/or additions of those initial hypotheses (Pawson & Tilley, 1997).

## 3 A realist evaluation of the CHW programme in Nigeria

Documentation of national Maternal, Newborn, and Child Health (MNCH) statistics in Nigeria started in early 1990s (ICF-International & NPC, 2014). Despite significantly reducing maternal and neonatal mortality by 60% and 50% respectively since 1990, these indices remain high at 814/100,000 and 37/1,000 births respectively, particularly in rural areas of the country, where vulnerable groups reside (FMoH, 2011, 2013; WHO, 2015).

In 2012, the Government of Nigeria (GoN) launched a Subsidy Reinvestment and Empowerment Programme (SURE-P), to invest revenues from fuel subsidy reduction into a social protection scheme for the benefit vulnerable populations, especially in rural areas of Nigeria. The SURE-P scheme had an MCH component (i.e. SURE-P/MCH) geared to improve the lives of mothers and their infants (NPHCDA, 2012). The SURE-P/MCH scheme comprised both supply and demand components. The supply component aimed to broaden access to quality maternity services and improve MCH outcomes through recruiting, deploying and training CHWs, infrastructural development and increasing availability of supplies and medicines. The demand component aimed to increase utilization of health services during pregnancy and at birth using a conditional cash transfer (CCT) programme. CCTs were given to pregnant women who register at a primary health care(PHC) centre, where they get health check-ups, deliver at a health facility and take their baby for the first series of vaccinations (Ebenso et al., 2015).

Since June 2015, a team of researchers from the University of Leeds, UK and the University of Nigeria have been using realist evaluation to assess the extent to which and under what circumstances, the SURE-P/MCH scheme (or CHW programme) promotes equitable access to quality services and improves MCH outcomes (Mirzoev et al., 2016). Methodology for the realist evaluation consisted of three inter-related steps: i) initial programme theory development, ii) theory validation and iii) theory consolidation. The first step aimed to develop the programme’s theory and the other two steps, to test it using empirical observations. Details of the three steps have been reported elsewhere (Mirzoev et al., 2016). Four months after commencing the evaluation (i.e. October 2015), the newly elected President of Nigeria reversed the policy on fuel subsidy reduction in order to catalyse the economic growth, thereby stopping government funding to SURE-P programme. Following discussions with our funders and Nigerian health authorities, it was decided that the best course of action was to implement the original methodology for the study, while also assessing the sustainability of achieved changes and the effects of ongoing advocacy efforts to entrench MCH in the national political agenda.

Subsequent sections of this paper reports the processes for and reflections on using logic modelling for the first evaluation step and how we explored context during the LM development.

## 4 Methods

**Fig. 2:**
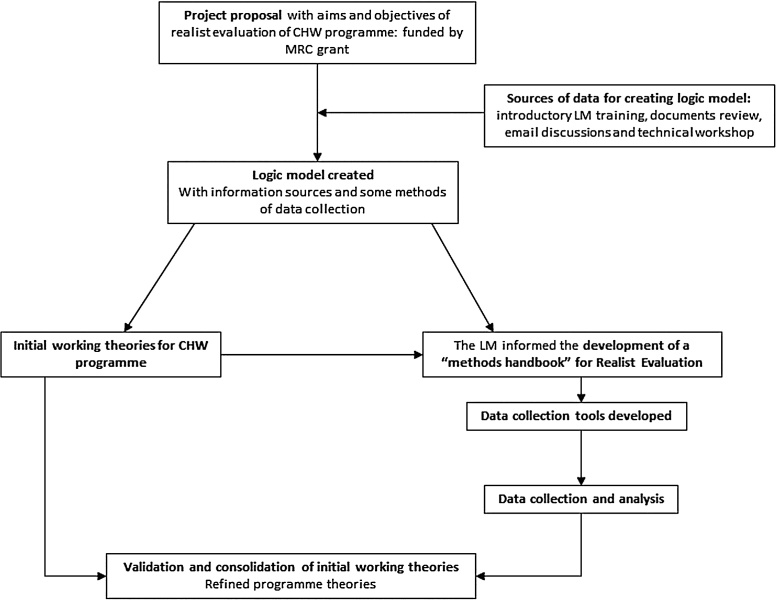
Position of logic modelling within realist evaluation of the CHW programme.

We now explain how we created the LM through a multi-stage process, using data from diverse sources. Fig. 2 depicts how the LM development for the CHW programme fits into the RE of the programme.

The figure shows that the sources of data used to develop the LM consisted of: i) an introductory LM training session, ii) documents review, iii) email discussions, and iv) technical workshop for researchers. The LM in turn informed development of initial working theories and a first version of the methodology handbook for the evaluation which was designed to be a living document that is updated as the evaluation progressed. At the time of writing this paper, the methodology handbook is guiding data collection and analysis of the RE exercise. The process of developing the LM is summarized next.

**Fig. 3:**
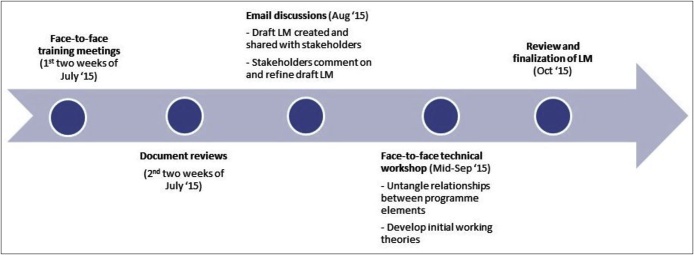
Process of developing a LM for CHW programme in Nigeria.

Linking back to purposes of LMs described in the background section, our LM served two purposes. First, to clarify the goals and conceptual gaps in programme stakeholders’ (i.e. researchers, policymakers and implementers) expectations of how the programme should work; and second, to inform measures of programme evaluation. Regarding the first purpose, logic modelling served to graphically represent stakeholders’ thinking of how the programme should work, by showing interrelationships between inputs, activities, outputs, outcomes and context. As no previous LMs were developed at inception and implementation stages of the CHW programme, the LM for this evaluation was used inductively as a tool for documenting the activities and outcomes the CHW programme hoped to achieve, and so retrospectively constructing the programme theory. Fig. 3 shows the process of creating the logic model took about three months from the training meetings in July 2015 through to finalization of LM in October 2015. This process is discussed next.

**Fig. 4:**
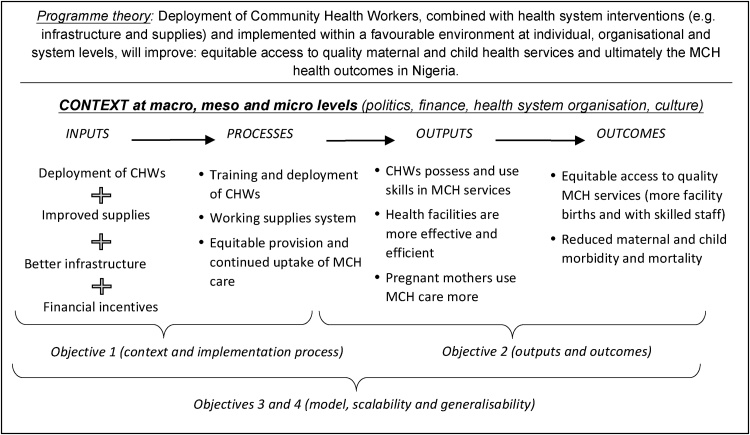
Conceptual framework for realist evaluation of CHW programme (Source: Mirzoev et al., 2016, pg5).
ii) **Documentary review:** Training meetings were followed by review of key documents, to extract relevant information about programme components from the documents. Documents were reviewed collaboratively by researchers in both institutions, who then shared their summaries with programme implementers and policymakers in Nigeria. The documents reviewed included health policies, the CHW programme implementation manual; and the national health management information system (NHMIS) policy, to identify the long-term vision and impact of the programme, and identify the inputs, activities, outputs and outcomes that were necessary for achieving these results (The-Presidency, 2012). Tentative contextual factors that can prevent/facilitate these results from happening were further refined.
iii) **Email discussions and teleconferences:** The draft LM created during steps i and ii was shared with policy makers and implementers to establish their understanding of the model. Programme implementers that contributed to the LM were: a) the national SURE-P programme manager; and b) oversight and validation (O&V) officers charged with monitoring SURE-P MCH activities at health facility level. The O&V officers produced monthly NHMIS reports on human resources; drugs and supplies; facilities upgrade; and expenditure by health facilities. As huge geographical distances prevented regular face-to-face meetings with policymakers and programme implementers, we used different information and communication technologies (M. S. Househ, Kushniruk, Maclure, Carleton, & Cloutier-Fisher, 2011) to support the interactions between researchers and programme stakeholders. Specifically, regular email discussions and monthly teleconferences with policy makers and implementers facilitated the iterative refinement of the draft model to ensure that it reflected stakeholders’ shared thinking of how the CHW programme should work in the context of Nigeria. Refining the LM involved clarifying and agreeing relationships among programme components. Consulting and engaging with local stakeholders ensured that only essential components of the multi-intervention CHW programme were represented in the LM. In other words, harnessing the tacit, formal and professional knowledge of stakeholders to identify essential components associated with how the CHW programme was intended to produce change, helped to narrow down the possible range of CMOs, so that a manageable number of mechanisms will be considered in the evaluation (Ling et al., 2012). The process of choosing the information to include in the LM was dynamic and iterative, and involved the addition of emerging/relevant components while simultaneously removing those deemed unnecessary for answering our evaluation questions. See sections 5.1 (Results) and 6.7 (Discussion and lessons learned) for more information on stakeholder consultation.
iv) **Technical workshop:** Building on the feedback from stakeholder consultations, we conducted a 3-day face to face technical workshop in September 2015, involving 10 researchers from universities of Leeds and Nigeria. The workshop was used to untangle relationships between programme elements, clarify contextual factors that affected the implementation and results of CHW programme, and subsequently develop initial working theories (IWTs) about how, why and in what circumstances the theories may work. The IWTs are summarized in the Results section. In the meantime, clarifying contextual factors that influenced the CHW programme entailed developing a number of matrixes to elicit tentative contextual factors. Data included in the matrixes were candidate theories of how the CHW programme should produce change; and a range of CMOs at micro, meso and macro levels that are related to the candidate theories. The matrixes facilitated systematic data extraction, critique and presentation of the CMOs. Table 2, Table 3 show examples of matrixes developed at the workshop, with Table 2 focusing on the supply and Table 3 on demand components of the programme. Content analysis of the matrixes aided prioritization of contextual factors for inclusion in the LM namely: political economy; policies; concurrent programmes; health system organisation; culture; and the values and perceptions of patients and health staff.

i) **Logic model training meetings:** Research teams from the University of Leeds and the University of Nigeria met in the first two weeks of July 2015 for a logic modelling training. The training used published literature on theory-driven, realist evaluation principles and logic modelling; and focused on how mapping programme inputs, activities and outcomes can help establish initial hypotheses for tentative relevant Contexts, Mechanisms, Outcomes and CMO configurations. Whilst different formats of logic models were considered as part of the training, a columnar and a multilayer format was chosen to facilitate the identification of inputs, activities, outputs and outcomes (Ziviani, Darlington, Feeney, & Head, 2011). Potential contextual factors that can influence the programme outputs and outcomes were identified and made more explicit by the participants. The process of identifying contextual factors started with eliciting a broad list of potential contextual factors that could influence the processes necessary to achieve inputs, outputs and outcomes in the CHW programme. Team consensus was achieved in line with the relevance of the evaluation questions, academic rigour and the presumed role in potential outcome patterns. Contextual factors were then organised at micro, meso and macro levels, using insight from the conceptual framework (see Fig. 4) for the realist evaluation (Mirzoev et al., 2016). Given this layered nature of context, we explored: a) the individual values and perceptions held by pregnant women and health workers i.e. micro context; b) the cultural norms that can influence the programme i.e. meso context; c) the institutional/health systems environment i.e. meso context; and d) policy and general societal factors i.e. macro context. Further explanation of how the above data was processed is provided below in section *iv) Technical Workshop”.*

**Table 2:** Matrix of tentative CMOs for supply component of the CHW programme.

**Candidate theory 1:** If different incentives (e.g. regular payments, training and improved working environment) are available in a timely manner, this will lead to improved and sustained health worker motivation, job satisfaction, performance and improved retention of staff in the context of Anambra State that is characterised by irregular salaries and poorly functioning facilities.		
Levels of Context	Levels of Mechanisms	Levels of Outcomes
**Individual context** C1 Non-experienced staff experience C2 Demoralized staff C3 Status and skill mix of MCH staff (CHWs, CHEWs, midwives)	**Individual mechanism** M1 Availability health workers and skill mix of MCH staff ensured	**Individual outcome** O1 Altruism and increased social responsibility O2 Increased staff motivation O3 Increased satisfaction O4 Improved staff performance
**Institutional Context** C3 Irregular salaries C4 Poorly functioning facilities C5 Strained working relationships between CHEWs and nurses following policy change in PHC facility management	**Institutional mechanism** M2 Continuous training of staff M3 Supportive supervision of staff M4 Collegial working environment M5 Regular payment are instituted M6 availability of equipment supplies and infrastructure	**Institutional outcome** O5 Increased staff retention O6 Improved quality of care delivered by facility O7 Increased utilization of ANC by women; O8 Increased skilled birth attendance.
**Macro Context** C6 New Government policy on social protection of vulnerable populations implemented as a pilot	**Macro mechanism** M7 Availability of SURE-P regulatory oversight	**Macro outcome** O9 Reduced maternal mortality rate O10 Reduced infant mortality rate
From the above, we can start formulating hypotheses such as: C1 + M1,M2,M4, M5 = O5,O6

**Table 3:** Matrix of tentative CMOs for demand component of the CHW programme.

**Candidate theory 2:** If communities in Anambra State (with poorly-functioning WDCs and irregular payment of incentives to women who are unaware of what MCH services are available), are mobilized and financially incentivized in a timely manner, this can lead to improved identification of women, increased coverage and improved utilization of MCH services.		
Levels of Context	Levels of Mechanisms	Levels of Outcomes
**Individual context** C1 Community members unaware of MCH services C2 CHWs are familiar with community context	**Individual mechanism** M1 Community members value sensitization messages to help them decide about using MCH services M2 CHWs build trusting relationships with pregnant women	**Individual outcome** O1 Individual empowerment of community members to demand services O2 Increased confidence in MCH services O3 Positive behaviour change reflected as increased utilization of services
**Institutional Context** C3 Poorly functioning WDCs C4 Irregular payment of incentives to WDCs	**Institutional mechanism** M4 Collective mobilization of WDCs M5 Regular payment of incentives to WDCs and CHWs/VHWs M6 Policymakers appreciate the need to invest in providing Mama kits to VHWs	**Institutional outcome** O4 Collective empowerment of WDCs O5 Improved identification of pregnant women
**Macro Context** C5 Inequitable geographical coverage of MCH services C6 Widespread poverty	**Macro mechanism** M7 Provision of high quality MCH services within rural communities M8 SURE-P regulatory oversight	**Macro outcome** O6 Increased utilization of services O7 Reduced maternal mortality rate O8 Reduced infant mortality rate

Next, we describe the resulting LM and explicate contextual factors and assumptions regarding how the CHW programme should lead to change, and present IWTs generated from the LM.

The purpose of prioritization was to narrow down the numerous ranges of CMOs to a manageable number to be considered for the evaluation.

## 5 Results

### 5.1 The resulting logic model and its components

**Fig. 5:**
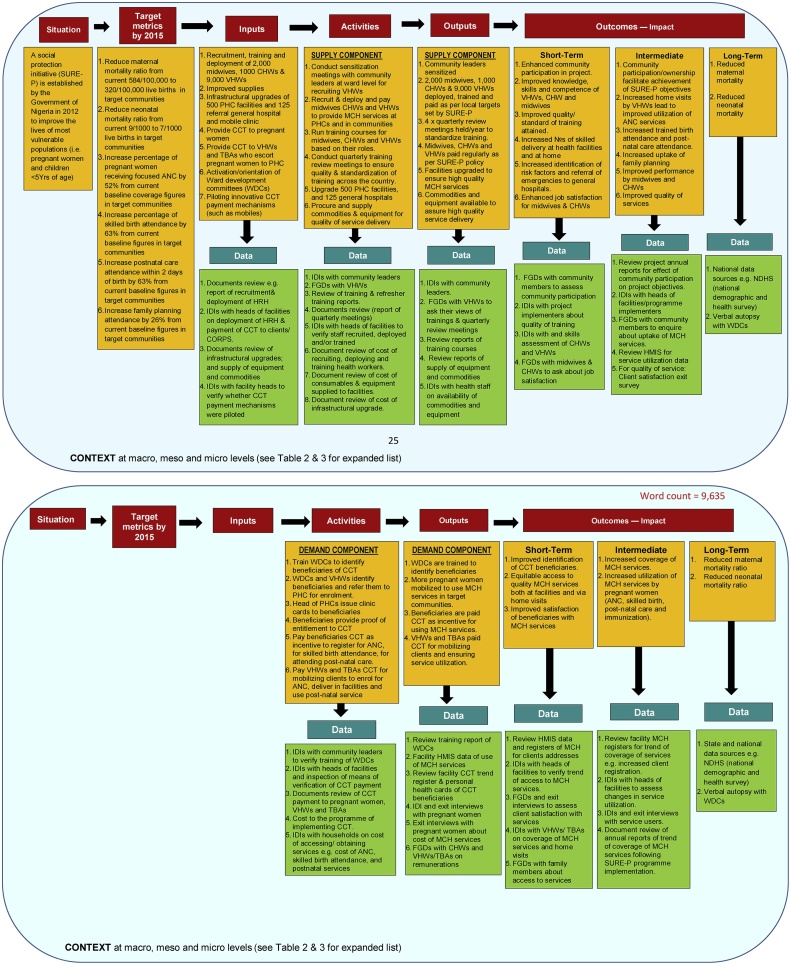
Logic model for the CHW programme in Nigeria.

The stakeholders of the CHW programme selected a modified W.K. Kellogg Foundation approach to logic modelling, hitherto used by the RUSH project for a bicycle helmet public information campaign (Moss, 2012; Williams, 2006; W.K.-Kellogg-Foundation, 2004). This LM format consists of columns stretching across the page from left to right, linked via connecting one-way arrows (see Fig. 5). The multi-level format includes three sections: an upper section portraying the target-input-process-output-outcome continuum; a middle section depicting various columns with information describing planned inputs, activities, outputs, and outcomes; and a lower section (i.e. data boxes) showing data collection methods and sources of information required for programme evaluation. This outcomes-based format was selected to facilitate a focus on each section of the LM to ensure that details of each column were systematically and easily summarised. However, as the process of elaborating programme components progressed, the difficulty of representing all relationships among the components of the CHW programme in a single, two-dimensional LM became apparent. Therefore, a series of inter-linked LMs were required to comprehensively unpack each intervention of the programme and capture interrelations among the supply and demand components of the CHW programme. The series of LMs required a five-page diagram (included as a supplementary file) to sequentially depict the interrelations among the supply and demand components of the CHW programme. However, due to the limitation of space, only a 2-page abridged version of our LM is presented in Fig. 5. The resulting LM comprised of six columns.

The first column (the situation that led to creation of the CHW programme) suggests the GON launched the SURE-P scheme following countrywide outcry to invest savings from Nigeria’s oil wealth into initiatives such as MCH that benefited vulnerable sections of the population. The column captures key programme contextual features: programme funder, programme aim, programme users and programme start date.

The second column shows specific programme objectives that reflect key target metrics. These relate to improvements in selected health and service delivery indicators such as maternal and neonatal mortality rates and utilisation of health services.

The third column relates to inputs invested in the programme to increase access to MCH services and improve MCH outcomes. The inputs in the figure can be further grouped into supply-related components (e.g. increased capacity of human resources and supplies) and demand-related components (e.g. community participation and provision of financial incentives to pregnant women to increase service uptake).

Column four captures groups of supply- and demand-side activities prioritized by the GON to facilitate delivery of quality MCH services in the context-specific implementation of the programme: i) sensitization meetings with community leaders to establish/ reactivate ward development committees (WDCs) to work with the health system to achieve planned outcomes; ii) deploying health staff to the programme; iii) running training workshops for WDCs and health workers; iv) renovating facilities and procuring necessary equipment and tools; v) paying CCTs to pregnant women and CHWs.

The fifth column (outputs), shows that the direct products of the activities implemented by the programme were measured in terms of the volume of work achieved (e.g., numbers of workshops conducted, health workers trained, and facilities renovated) and the number of customers reached (e.g., pregnant women who attended antenatal clinics, and who delivered in health facilities).

The last column (outcomes-impact), summarises the intended benefits or changes in the programme’s target population, further categorized as short-term, intermediate and long-term outcomes, with short-term outcomes leading to intermediate outcomes that, in turn, lead to long term-outcomes. Short-term outcomes of the programme included: increased individual knowledge, capacity and confidence of pregnant women to make decisions about their health; improved quality of training of health workers; and enhanced job satisfaction of workers. Intermediate outcomes included: making healthy choices and behaviours (e.g. uptake of family planning), and increased service user satisfaction and confidence in MCH services. Long-term outcomes included: lower maternal and neonatal mortality achieved through increased utilization of services in health facilities, and equitable access to health services.

The LM served as a focal point for discussions about developing evaluation measures by displaying where (i.e. data sources) and how (i.e. collection methods) to obtain information needed to determine the achievement (or not) of programme results. Data requirements were identified for all steps in the inputs-outcomes continuum and linked to the specific demand- or supply-side activities, outputs and outcomes by adding an extra row (see green boxes in Fig. 5) below each of the components. Data sources for assessing the programme included e.g., reports of trainings, NHMIS data and reports of national demographic and health surveys, while data collection methods included documents review, in-depth interviews, focus group discussions and verbal autopsies. We have listed data sources and data collection methods together in Fig. 5, due to the limitation of space in the data boxes.

Once the different features and sections of the LM were identified, the assumptions and contextual factors that can prevent the achievement of programme results were made explicit. Programme assumptions comprised of stakeholders’ beliefs about the programme and its inputs, including their perceptions of how the CHW programme will produce change (CDC, 2013). Although these are not explicitly shown in Fig. 5, the key assumptions about the CHW programme, extracted from the documents review, and stakeholder experiences were that:

i) GON’s financial support will be secure throughout the course of the programme
ii) Payment of financial incentives to pregnant women will stimulate positive behaviour change
iii) Health workers with the necessary capabilities will be recruited and deployed
iv) Knowledge and skills gained from training courses will be used to improve quality of care
v) Financial and non-financial incentives to health workers will stimulate staff motivation and retention.

In realist evaluation, contextual factors describe both the internal environment in which the programme operates and external factors that interact with and influence implementation, participation, and the achievement of outcomes of the programme (CDC, 2013). The exchange between researchers and programme stakeholders in creating the LM facilitated the identification of contextual factors that can potentially affect the production of intended programme outcomes or unintended outcomes. As mentioned in the Methods section, CMO matrixes were then developed at a technical workshop in September 2015, to further clarify and systematically elicit contextual factors that arose from engagement with policymakers and programme implementers. Finally, content analysis of the matrixes led to the prioritization of following 6 groups of contextual factors at macro level (policy and societal), meso level (health facilities, communities) and micro levels (individual staff and patients) for inclusion in the LM namely:

i) Political events that influenced policy and programme activities (macro context).
ii) Other MCH initiatives implemented by non-governmental organizations (macro context).
iii) Organization of the Nigerian health system and health services (meso context)
iv) Cultural norms that influence programme outcomes in reaching pregnant women (meso context)
v) Socioeconomic factors of pregnant women which affect their ability to pay for and/or utilize MCH services (micro context)
vi) Individual values and perceptions which affect health-seeking behaviour and service provision (micro context)

In Fig. 5, the context is depicted as a blue background around the LM, with a statement of factors that interact with/influence the CHW programme. Used in this way, the CMO matrixes: a) were effective tools for eliciting and incorporating context into the LM, and b) enhanced the LM development process and the final LM.

Our study follows the hypothesis that the nature of the issue that needs improvement (i.e. MCH in Nigeria) and the context in which the programme operates influence the ways in which a particular policy instrument evolves and operates (Manzano-Santaella, 2011). Context was further taken into consideration for developing initial working theories as part of CMO configurations that emerged from using the LM to map the components for this large-scale programme.

### 5.2 How the LM informed development of initial working theories

Initial working theories (IWTs) or candidate programme theories can be used to accomplish two objectives. First, to identify components of a programme needed to solve a problem and achieve expected outcomes (Davidoff, Dixon-Woods, Leviton, & Michie, 2015). Second, to identify a programme's theory of change i.e. assumptions about mechanisms that link a programme's processes and inputs to outcomes (Weiss, 1995). Our LM helped to accomplish the first objective, and the IWTs addressed the second. As mentioned earlier, we used the technical workshop in September 2015 to untangle relationships between elements/components of the CHW programme, to develop matrixes for eliciting contextual factors and for developing IWTs. The matrixes developed during that workshop (refer Table 2, Table 3) served as the dataset for developing and refining IWTs. Specifically, some ‘if…then’ statements of change were developed and subsequently converted into IWTs. One example of an ‘if…then’ statement of change identified at the workshop is: “*If local communities are mobilized and supported to participate in the CHW programme, then participating communities will have increased capacity to identify and refer more pregnant women to hospitals that provide quality maternity care”*. The ‘if…then’ statements had a key implicit contextual purpose: i.e. to clarify what will happen if the assumptions/theories were not implemented in this way for all programme users in all institutional contexts.

Similarly, we present two examples of IWTs that emerged from converting ‘if…then’ statements into programme theories. The first example is drawn from the supply component of the CHW programme to illustrate contextual factors that can influence health worker motivation, performance and retention. The second example, from the demand component, illustrates contextual factors that can influence service utilization by local communities:

i) Providing timely financial and non-financial incentives (such as regular salaries, training and improved working environment) (Cs) to health workers, will make staff feel valued (M), leading to improved staff motivation (O), job satisfaction (O), performance and retention (O) in the context of Anambra state that is otherwise characterised by irregular salaries and poorly-functioning facilities (Cs).
ii) Mobilizing the ward development committees (WDCs) and providing financial incentives to local communities (Cs) will stimulate WDCs to search for pregnant women who require referral (M), thus leading to improved identification and referral of pregnant women (O), increased coverage (O) and utilization of MCH services (O) in the context of Anambra State, which is otherwise characterized by inactive WDCs and poor uptake of available MCH services (Cs).

From the foregoing, IWTs enabled us to infuse the ‘if…then’ statements with implied contexts (Cs), that may enable/disable assumed programme mechanisms (Ms) and consequently result in planned/unplanned outcomes (Os) that could be empirically verified during the evaluation. The list of ITWs identified at the workshop were later reviewed and endorsed by programme implementers and policymakers in Nigeria. The endorsed working theories (available as supplemental material) will be refined and validated as part of data collection in ongoing realist evaluation of the programme.

## 6 Discussion and lessons learned

In this paper, we reflected on how other scholars explored and incorporated context into their LM during theory-driven evaluation. We also shared how we developed a LM within an ongoing realist evaluation of a CHW programme in Nigeria, which aimed to assess the extent which and under what circumstances the CHW programme promoted equitable access to maternity services and improved health outcomes. As no previous LMs were developed at the inception stage of the CHW programme, we used the LM to clarify programme goals, map interrelations among programme elements and develop evaluation measures to verify how (i.e. intervention processes), in what circumstances (i.e. contextual factors), for whom and why the CHW programme achieved or did not achieve its outcomes (Westhorp, 2014).

After using an adapted ATM approach and context map to depict their programmatic assumptions (i.e. mechanisms of change) in context, Renger et al. (2015) posited that the utility of context maps (refer Fig. 1) can be augmented by applying context maps from a realist evaluation perspective to enhance the ability of evaluators to explore and interpret how different contextual factors may influence a programme’s mechanism(s) of change, and how different mechanisms of change can in turn, alter programme outcomes. In line with their proposal, we developed our logic model from a realist evaluation perspective by elaborating different programmatic assumptions and contexts (at macro, meso and micro levels) that can affect outcomes of the CHW programme. Moreover, we used CMO matrixes as a tool to systematically elicit, analyse and prioritize contextual factors that were incorporated into the LM. Finally, the CMO matrixes helped us to infuse context into initial working theories (see previous section) as part of CMO configurations that emerged from using the LM to map the components for the multi-intervention CHW programme.

We share and briefly discuss the following eight lessons learned from the process of creating a LM within our realist evaluation, to inform future similar efforts:

### 6.1 Logic model format, features and process should be approached with flexibility

Our experience of creating a LM for the CHW programme showed that different versions of logic models may be needed during the development process, at different evaluation stages and across projects. Creating a LM is not an end in itself. By displaying inter-relations among programme components, creating a LM is an essential step in the task of developing an empirically-based and theoretically-grounded *model* of complex relations between a programme, its outcomes and its broader context. Involving policy makers and implementers in creating the LM facilitated better understanding of the logic that connected the CHW programme to its outcomes (Ling et al., 2012), thus minimizing the risk that external researchers will have only a limited knowledge of the local context.

Involving local stakeholders also minimized the danger that realist evaluation will be unable to distinguish between a failed theory and failed implementation of the programme (Ling et al., 2012). As shown in the previous section, the process of developing LMs can also facilitate the identification of candidate theories (or hypotheses) for CMOs and corresponding CMO configurations of how the CHW programme should produce change. Lastly, LMs can inform the development of measures of evaluation (see Fig. 2) that are linked to expected programme outputs, to guide data collection and analysis in the realist evaluation. Mason and Barnes (2007) claim that detailed logic models depicting programme theories can only be obtained over time, through immersion and interaction with stakeholders and that initially-drafted logic models always lack cumulative learning and should be treated as such. Therefore, as more sophisticated understandings of the CHW programme are developed, it is expected that our LM will be updated (Baum et al., 2014).

### 6.2 The purpose of logic model should be made explicit

We acknowledge that programme stakeholders come to the LM development process with their own, often different, expectations of aims, objectives and approaches. Whilst the flexibility of the logic modelling process allows for a variety of perspectives and approaches to be taken within a programme’s context, nevertheless, stakeholders should understand the purpose for which they are developing their LM. In our case, the LM helped to clarify the goals of the CHW programme, map interrelations among various elements of the programme and develop measures of evaluation. Petersen et al. (2013) argue that, in the absence of LMs, evaluators may be tempted to design data collection systems that focus exclusively on programme outcomes, neglecting implementation analyses that measure the processes needed to achieve identified outcomes or partially account for the context in which interventions take place. It should be expected that in the majority of programmes, at the outset, a programme’s theory will very likely be “under-developed, in conflict with real conditions, and unclear” (Chatterji, 2016). From our experience, a clear and agreed purpose for logic modelling is an important premise to start the process of elucidating and developing those theories.

### 6.3 Documentation and stakeholder involvement facilitate development of LMs

Developing a coherent LM requires in-depth understanding of a programme and its components—information that can often be found in programme manuals and plans. This is especially so for programmes such as the CHW programme, for which no prior LMs were created at the programme inception stage. In our experience, the availability of a well-designed programme manual (NPHCDA, 2012) and a comprehensive national NHMIS policy (FMOH, 2006) facilitated our understanding of the vision, goals and objectives of the CHW programme. The absence of such documents would make it partly difficult to articulate and represent underlying theories of how the programme should work, or to discuss them with stakeholders. However, in situations where programme documents are sketchy or unavailable, scholars may use relevant information from initial primary data collection e.g., stakeholder meetings, individual interviews and focus group discussions to create LMs. Leeuw (2003) argued that an evaluator-guided, empirical-analytical approach to modelling and testing assumptions may not be enough to construct programme theory models and that a number of other methods should be used by evaluators to adequately grasp problems. This may include the elicitation of mental models to predict behaviours of decision makers and action-takers in organizations to postulate theories of action and change.

### 6.4 LM development is an iterative, ongoing and time-consuming process

It took our research group approximately 12 weeks, i.e. from the training meetings in July through to finalizing the model in October 2015, to create the LM for the CHW programme. It is fair to emphasize that while programme documentation are important, they are not enough by themselves. Additional to document reviews mentioned above, data for creating our LM were derived from face-to-face meetings of researchers, monthly teleconferences and email discussions with stakeholders, and technical workshop with researchers. This supports the arguments of Kaplan and Garrett (2005), that developing robust LMs for complex intervention programmes is not a quick and easy process. Helitzer et al. (2009) also argue that poorly-designed LMs that are often based on a rushed process, characterized by a lack of attention to local context and stakeholder views, or have overly simplistic assumptions which are superficial and unhelpful for monitoring and evaluating programmes.

### 6.5 LMs may fail to capture programme complexity and contextual nuances

LMs typically depict linear and simplified relationships (depicted by sequential arrows at the upper section of Fig. 5) between inputs, activities and outputs, or between outputs and outcomes. However, in reality, interrelationships between and among inputs, activities, outputs, and outcomes are more complex (Renger et al., 2015; Rogers, 2008) due to micro, meso and macro contextual factors. We observed that as the process of refining the draft LM for the CHW programme progressed, it was difficult to represent all relationships among programme elements in a single, A4-sized, two-dimensional LM format. A five-page series of LMs were subsequently required to unpack each intervention of the CHW programme and capture complex interrelations among the various components of the multi-intervention programme. However, due to constraints of space, we can only share an abridged (two-page) version of the LM for the CHW (see Fig. 5). The full version of the LM has been uploaded as a supplementary file. Petersen et al. (2013) maintain that although it may seem overwhelming to fully specify all relevant linkages within complex programmes, nevertheless, articulating a LM or a series of LMs can help evaluators distil programme interventions to their core elements. If researchers will go down this route, it would be important to identify any possible points of connection of different LMs to ensure that the overall big picture is in sight. This echoes Chatterji’s (2016) views that although, the logic underlying comprehensive programmes in the minds of some key stakeholders is linear, mimicking that of RCT designs, the real conditions defy linearity because the causal pathways to outcomes are vague, disconnected and unstable.

### 6.6 In realist evaluation, LM can be used a tool for identifying tentative contexts, mechanisms and outcomes

Although scholars have identified that a limitation of simple logic models (e.g. table-format LM), is their failure to capture the context in which a programme operates (Morell, 2014; Renger et al., 2015; Rogers, 2008), evaluators can address this limitation by either adopting more complex LM formats or applying a realist evaluation perspective to logic modelling. LM development can be used as a tool for identifying initial hypotheses for relevant tentative contexts, mechanisms and outcomes and CMO configurations of how the programme will produce change. Taking a collaborative approach to elaborating the components of a programme on a LM and refining the LM might imply that several variations in and versions of the LM are produced before stakeholders eventually validate and approve for the LM for realist evaluation of the programme.

### 6.7 The process of developing LM itself can facilitate closer links with stakeholders

Creating LMs typically requires engaging with a range of stakeholder groups from within and outside the programme e.g. planners, funders, implementers, service users and policymakers. The co-production process enabled us to forge partnerships with stakeholders who were geographically dispersed across two continents (Europe and Africa). While Kaplan and Garrett (2005), p169) argue that using LMs to foster collaboration can be challenging for organizations whose members are spread widely in terms of geographical location, in our experience, huge geographical distances were bridged by the availability of information and communication technologies (ICTs) such as internet connections and the use of emails, teleconferences and webinars, that enhanced communication and exchange of ideas in a research-policy partnership (Mirzoev et al., 2012). Although budget constraints affected our ability to hold regular face-to-face meetings where all stakeholders were physically present, nonetheless, the availability of mobile telephones, email and internet connections specifically enhanced the level of interaction and facilitated partnership building among policymakers, implementers and researchers. Furthermore, the availability of voice and video conferencing technologies increased the level of social presence of stakeholders during LM discussions. These range of ICTs ensured continuity of communication at a distance and facilitated closer links among stakeholders. Besides ICT, we found that embedding our research within the policy and practices of the Ministry of Health (MOH) Nigeria (COMDIS-HSD, 2012) facilitated collaboration of researchers with policymakers and with programme implementers. As part of the embedding process, policymakers and implementers in Nigeria were involved as collaborators at every stage of the study. For example, the national programme manager of the CHW programme contributed to research design as well as to identification and prioritization of context-specific research questions during the research conception phase (from October 2013 to January 2014). Similarly, policymakers and implementers contributed to development of the LM, which informed data collection phase of the evaluation; and will contribute to data analysis and help to refine and validate programme theories. The primary objective of involving stakeholders at all stages of the study was to increase trust in the research-policy partnership (WHO, 2012) and enhance ownership of the research (Ghaffar et al., 2017). Whereas Wong (2009), regards mutually trusting relationships as a key facilitator of collaborative research, Kothari, Hovanec, Hastie, and Sibbald (2011) maintain that having a “champion” within an organization who supports co-development of research is essential for embeddedness. In our experience, a combination of factors that promoted embeddedness were: i) a national programme manager that championed the embedding process, ii) a pre-existing relationship with the programme manager, as a number of our co-authors (BSC, EE, NE, OO) had worked with the manager on a previous project; iii) the use of different ICTs to bridge geographical distances between researchers in the UK and collaborators in Nigeria (Kothari et al., 2011).

### 6.8 Logic mapping provided a shared language for understanding the programme and strengthening stakeholder learning

Logic mapping provided an opportunity and a shared language for understanding programme nuances, contextual constraints and facilitators and strengthening stakeholder learning. Knowlton and Phillips (2012) argue that, because LMs enhance learning through the iterative exchange of information and experience, they offer important features to organizations that value evidence, diversity, dialogue, feedback and systematic planning. In our experience, two key factors enhanced the shared language among stakeholders dispersed across continents. First, the LM training meetings (of July 2015) facilitated by an expert in realist methodology (AM) fostered shared understanding among researchers, of the concept of logic modelling and how to develop a LM for the CHW programme. We built on the shared understanding among researchers, by using the technical workshop in September 2015, to co-produce CMO matrixes and prioritize contextual factors for inclusion in the LM. As outlined in Section 5.2, the CMO matrixes subsequently served as the dataset for IWT development. Secondly, the regular use of ICTs (email discussions and monthly teleconferences) made it easier to coordinate the process of elaborating programme components for the LM development. Furthermore, the collaborative approach adopted for developing and refining the draft LM also provided our stakeholders (policymakers, implementers and researchers) with a shared language and an approach for understanding the programme nuances. Stakeholders reported that using the LM to explicitly depict programme inputs, activities, outputs and outcomes helped them to better appreciate the range of resources needed by the programme and potential contextual constraints to clarify previously implicit assumptions of how the CHW programme was perceived to work. Our findings support Househ et al.’s research (2011) that reported that a range of strategies including face-to-face meetings and different ICTs such as audio and web conferencing may be required to facilitate knowledge exchange processes and indirectly promote a shared language of among collaborating partners.

## 7 Conclusion

Multi-intervention health programmes such as the CHW programme in Nigeria are complex, dynamic and always evolve in response to local contexts, service user preferences and other events that can affect the implementation and impact of the interventions. As complex intervention programmes are difficult to evaluate by traditional experimental designs, we applied a programme logic model as part of an ongoing realist evaluation project, to help depict the relationships among the inputs, activities, outputs and outcomes of the CHW programme. We also used logic model development as a tool for identifying initial hypotheses for relevant tentative contexts, mechanisms and outcomes and CMO configurations of how the programme will promote equitable access to quality maternity services and improved health outcomes. In our experience, adopting a realist evaluation perspective to logic modelling helped us explore different programmatic assumptions (i.e. mechanisms of change) and contexts (at macro, meso and macro levels) that could affect outcomes of the CHW programme. In this sense, logic models can be adequate tools for addressing complexity but they need to be understood as iterative tools to accumulate learning that are always imperfect, never linear and should be constructed with multiple-methods and flexible formats.

## Authors' contributions

BE, AM, TM and RH jointly conceived the study; BE led data collection and the writing of this paper with contributions from TM, EE, AM, BU, AM, OO, RH, NE, JH, JN, TE. All authors read and approved the final version of the manuscript.

## Funding

This work was supported as part of the Joint MRC/ESRC/DFID/ Wellcome Trust health systems research initiative (grant ref: MR/M01472X/1). None of the funders had any role in the design of this study.

## Ethics approval and consent to participate

Ethical approval for the wider study were obtained from the School of Medicine Research Ethics Committee at the Faculty of Medicine and Health at the University of Leeds (ref: SoMREC/14/097) and the Health Research Ethics Committee at the University of Nigeria Teaching Hospital (ref: NHREC/05/02/2008B-FWA00002458-1RB00002323).

## Competing interests

The authors declare that they have no competing interests

## Footnotes

1 **List of Abbreviations:** CHW = Community Health Worker; GON = Government of Nigeria; MCH= Maternal and Child Health; PHC= Primary Health Care; SURE-P = Subsidy Reinvention and Empowerment Programme; VHWs = Village Health Workers; WDCs = Ward Development Committees.
