# Supplementary figures and images for: Dealing with context in logic model development: Reflections from a realist evaluation of a community health worker programme in Nigeria
Source: Eval Program Plann. 2019 Apr;73:97–110. doi: 10.1016/j.evalprogplan.2018.12.002 (PMC6403102; doi:10.1016/j.evalprogplan.2018.12.002)

## Appendix 2: Logic model for using the CHW programme to improve maternal & child health in Nigeria

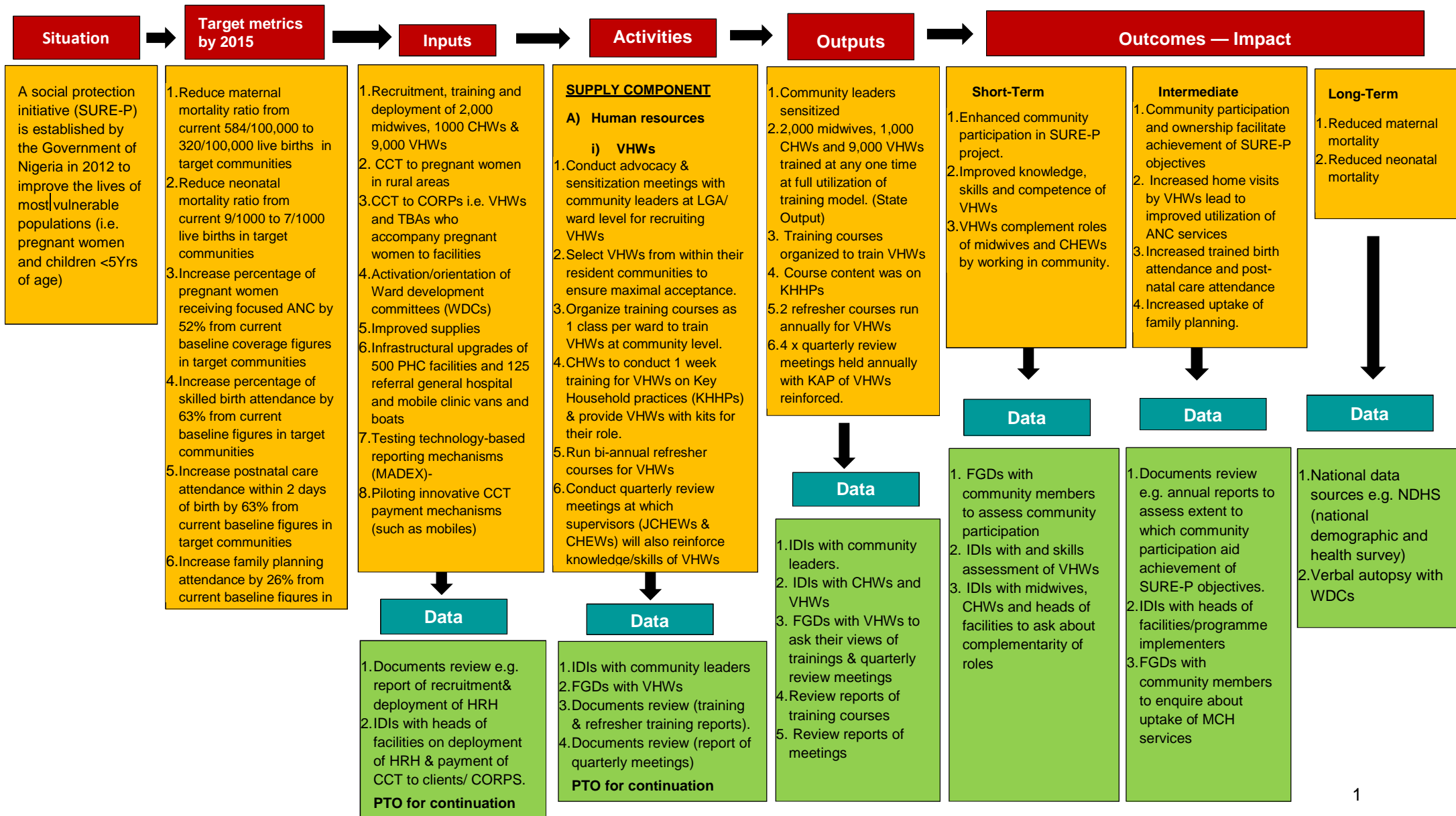

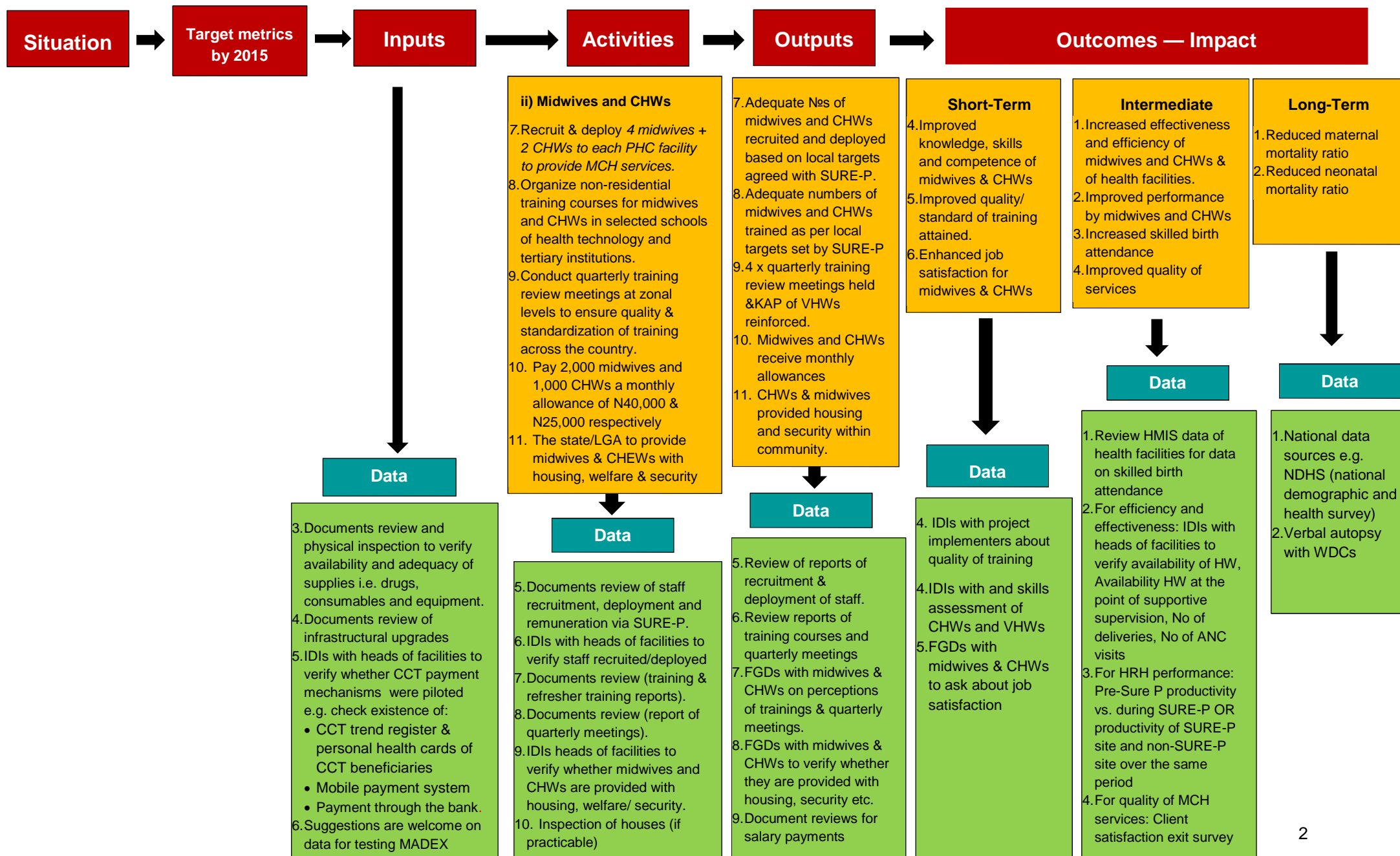

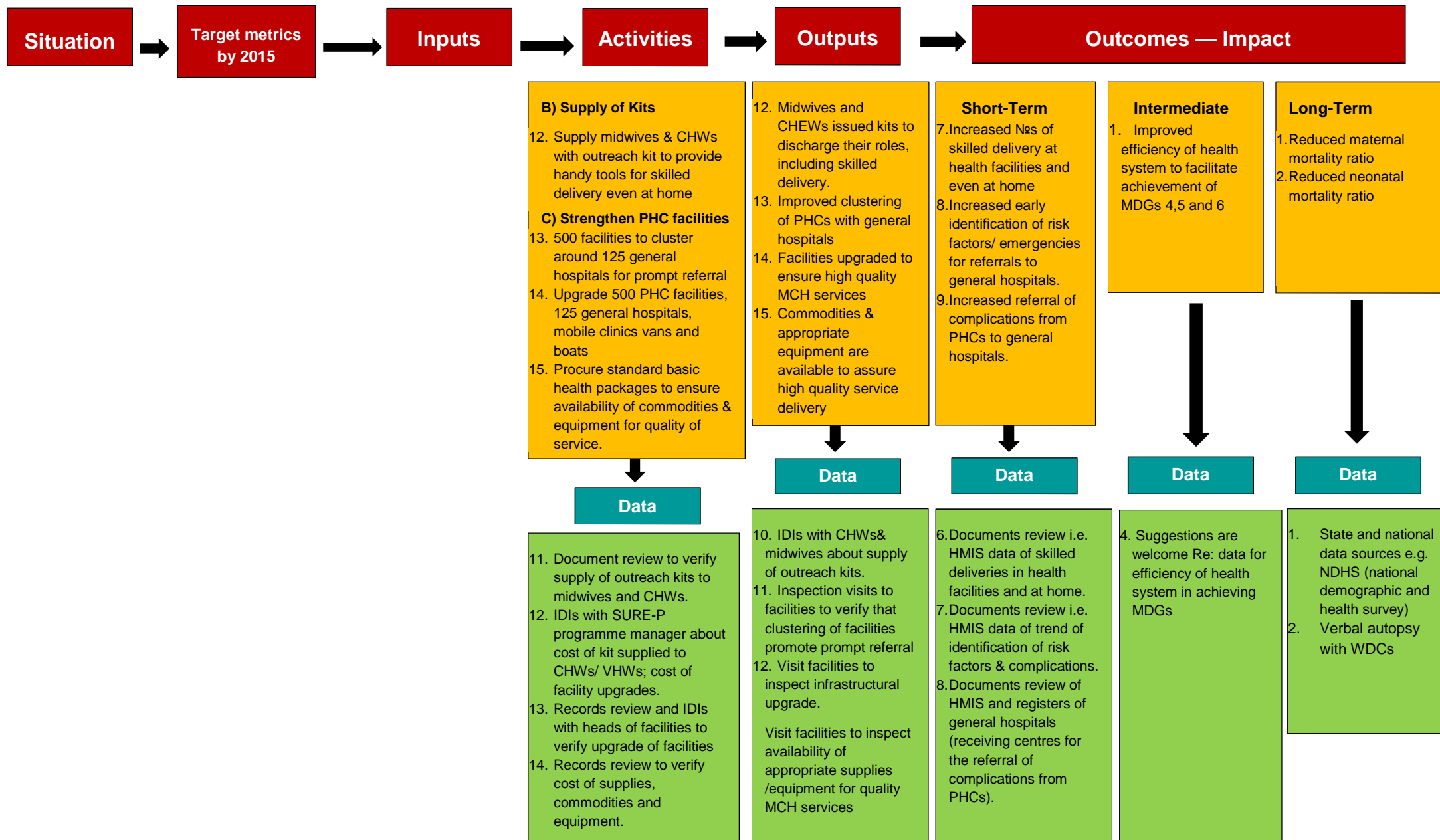

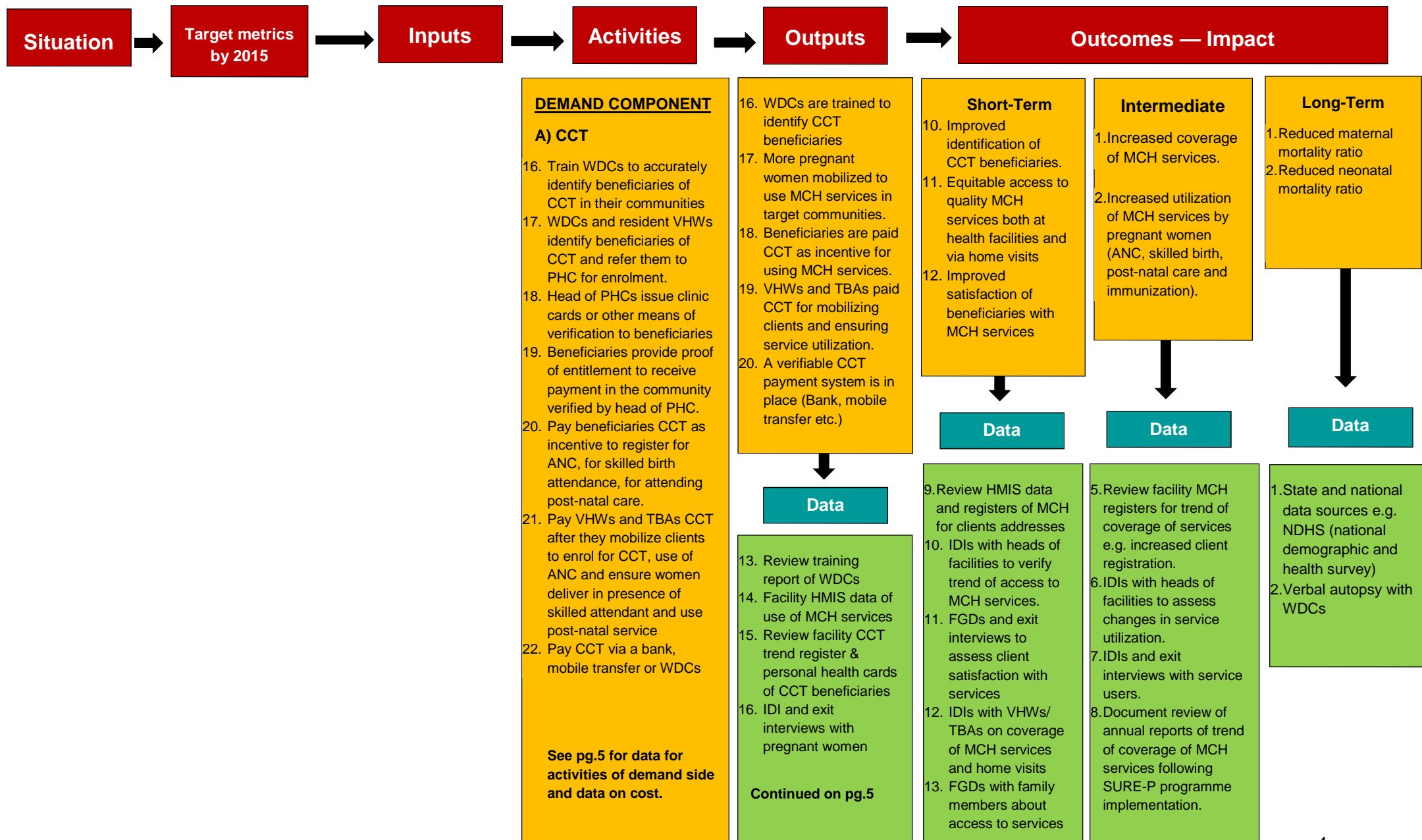

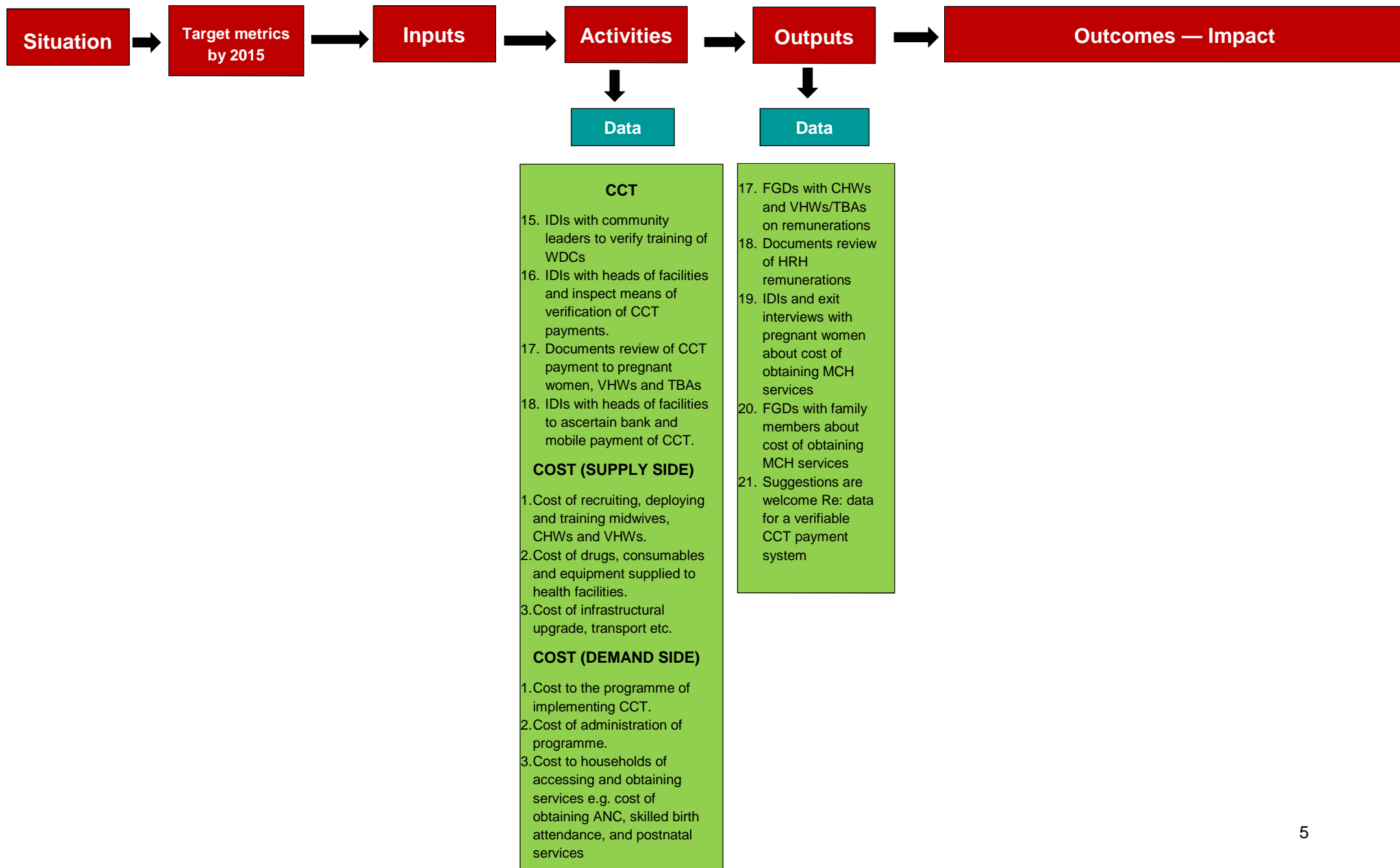

Supplement: Supplementary file 1 [file mmc1.pdf]
